# Stereoselective synthesis, X-ray analysis, computational studies and biological evaluation of new thiazole derivatives as potential anticancer agents

**DOI:** 10.1186/s13065-018-0420-7

**Published:** 2018-05-11

**Authors:** Yahia N. Mabkhot, Mohammed M. Alharbi, Salim S. Al-Showiman, Hazem A. Ghabbour, Nabila A. Kheder, Saied M. Soliman, Wolfgang Frey

**Affiliations:** 1 Department of Chemistry, College of Science, King Saud University, P. O. Box 2455, Riyadh, 11451 Saudi Arabia; 2 Department of Pharmaceutical Chemistry, College of Pharmacy, King Saud University, P. O. Box 2457, Riyadh, 11451 Saudi Arabia; 3 Department of Medicinal Chemistry, Faculty of Pharmacy, University of Mansoura, Mansoura, 35516 Egypt; 4 Department of Chemistry, Faculty of Science, Cairo University, Giza, 12613 Egypt; 5 Department of Pharmaceutical Chemistry, Faculty of Pharmacy, King Khalid University, Abha, 61441 Saudi Arabia; 6 Department of Chemistry, Rabigh College of Science and Art, 344, Rabigh, 21911 Saudi Arabia; 7 Department of Chemistry, Faculty of Science, Alexandria University, P.O. Box 426, Ibrahimia, Alexandria, 21321 Egypt; 8 Institut für Organische Chemie, Universitӓt Stuttgart, Pfaffenwaldring 55, 70569 Stuttgart, Germany

**Keywords:** Thiazoles, X-ray crystallography, Computational studies, DMF-DMA, Cytotoxic activity

## Abstract

**Background:**

The synthesis of new thiazole derivatives is very important because of their diverse biological activities. Also , many drugs containing thiazole ring in their skeletons are available in the market such as Abafungin, Acotiamide, Alagebrium, Amiphenazole, Brecanavir, Carumonam, Cefepime, and Cefmatilen.

**Results:**

Ethyl cyanoacetate reacted with phenylisothiocyanate, chloroacetone, in two different basic mediums to afford the thiazole derivative **6**, which reacted with dimethylformamide- dimethyl acetal in the presence of DMF to afford the unexpected thiazole derivative **11**. The structures of the thiazoles **6** and **11** were optimized using B3LYP/6-31G(d,p) method. The experimentally and theoretically geometric parameters agreed very well. Also, the natural charges at the different atomic sites were predicted. HOMO and LUMO demands were discussed. The anticancer activity of the prepared compounds was evaluated and showed moderate activity.

**Conclusions:**

Synthesis of novel thiazole derivatives was done. The structure was established using X-ray and spectral analysis. Optimized molecular structures at the B3LYP/6-31G(d,p) level were investigated. Thiazole derivative **11** has more electropositive S-atom than thiazole **6**. The HOMO–LUMO energy gap is lower in the former compared to the latter. The synthesized compounds showed moderate anticancer activity.
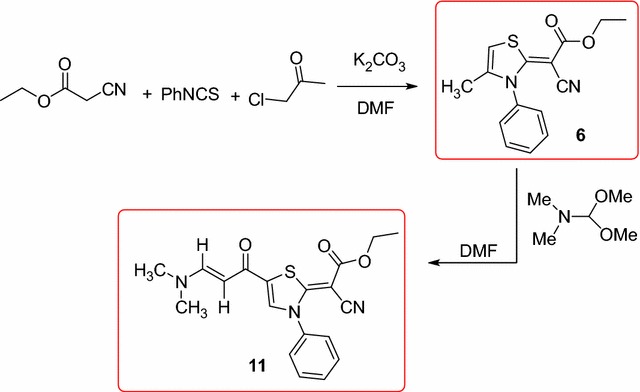

**Electronic supplementary material:**

The online version of this article (10.1186/s13065-018-0420-7) contains supplementary material, which is available to authorized users.

## Introduction

Currently marketed anticancer medications have increasing problems of various toxic side effects and development of resistance to their action. So, there is an urgent clinical need for the synthesis of novel anticancer agents that are potentially more effective and have higher safety profile. The synthesis of different thiazole derivatives has attracted great attention due to their diverse biological activities that include anticonvulsant [1, 2], antimicrobial [3, 4], anti-inflammatory [5, 6], anticancer [7], antidiabetic [8], anti-HIV [9], anti-Alzheimer [10], antihypertensive [11], and antioxidant activities [12]. The reaction between active methylene compounds with phenylisothiocyanate and α-haloketones in DMF in the presence of potassium hydroxide is the simple and convenient method for the synthesis of many thiazole derivatives [13–15]. In continuation of our interest in the synthesis of new biologically active heterocyclic rings [16–22] and motivated by these information, it was thought worthwhile to synthesize some novel thiazole derivatives and to test their antitumor activity in order to discover new potentially biologically active drugs of synthetic origin.

## Results and discussion

### Chemistry

The thiazole derivative **6** was previously obtained by the reaction of ethyl cyanoacetate with phenylisothiocyanate and propargyl bromide in DMF-NaH [23]. The presence of many functional groups attached to this bioactive thiazole ring motivated us to prepare it again to use it as a precursor for some new heterocycles bearing the bioactive thiazole ring. In this research, we used, instead of propargyl bromide, other reagents, such as chloroacetone, and we studied the configuration of the isolated products.

**Fig. 1:**
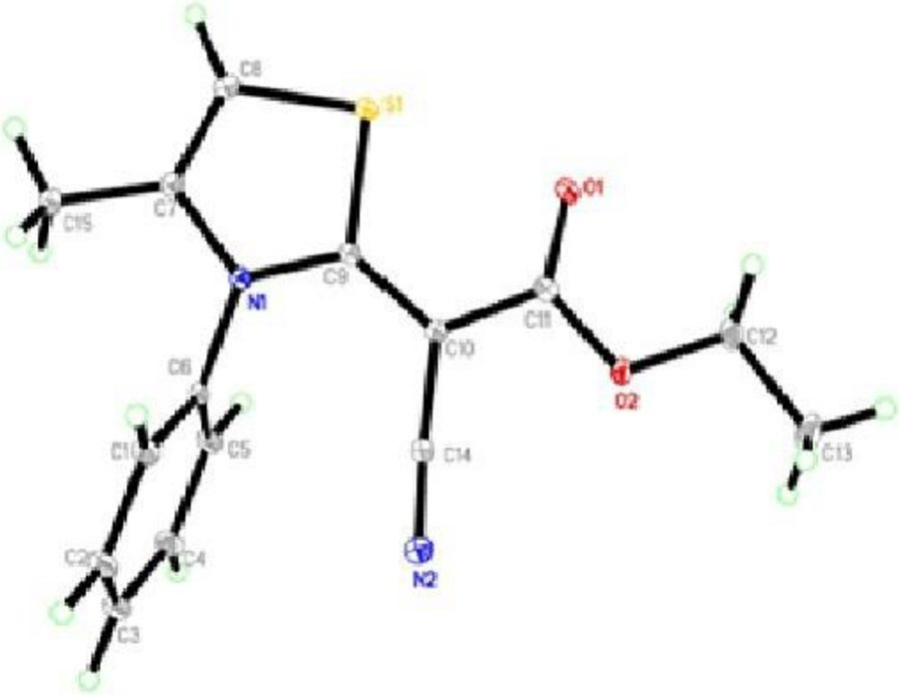
ORTEP diagram of the thiazole **6**. Displacement ellipsoids are plotted at the 40% probability level for non-H atoms

**Scheme 1:**
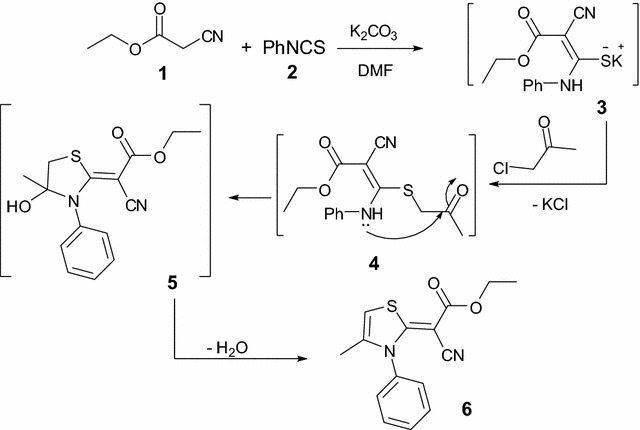
Synthesis of (*Z*)-ethyl 2-cyano-2-(4-methyl-3-phenylthiazol-2(3*H*)-ylidene) acetate (**6**)

The reaction of ethyl cyanoacetate with phenylisothiocyanate and chloroacetone in DMF-K_2_CO_3_ or sodium ethoxide solution afforded only one isolable product. The isolated product was identified as (*Z*)-ethyl 2-cyano-2-(4-methyl-3-phenylthiazol-2(3*H*)-ylidene) acetate (**6**). Its structure was established from X-ray analysis (Fig. 1) [24] and was confirmed using elemental and spectral analysis (IR, ^1^H NMR, ^13^C NMR). The suggested mechanism for the synthesis of thiazole **6** is outlined in Scheme 1.

**Fig. 2:**
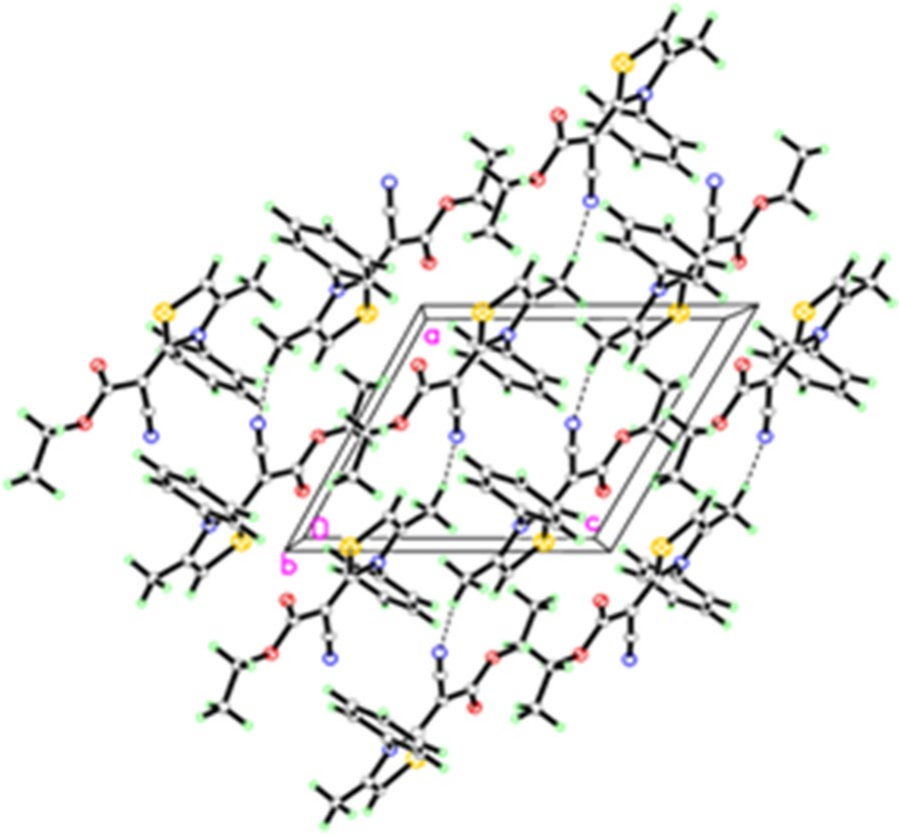
Molecular packing of thiazole **6** viewed hydrogen bonds which are drawn as dashed lines along *a* axis

The configuration of thiazole **6** was confirmed using X-ray analysis (Figs. 1, 2).

**Scheme 2:**
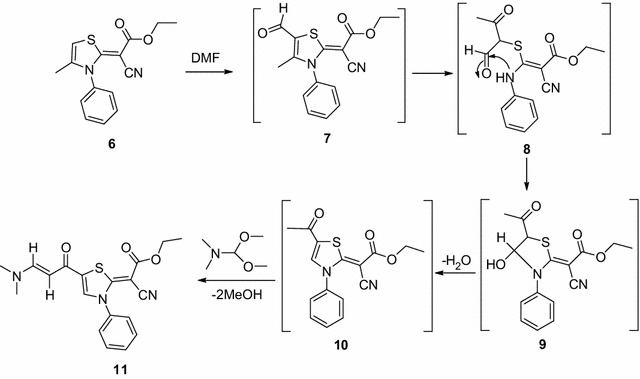
A suggested mechanism for the synthesis of thiazole derivative **11**

**Fig. 3:**
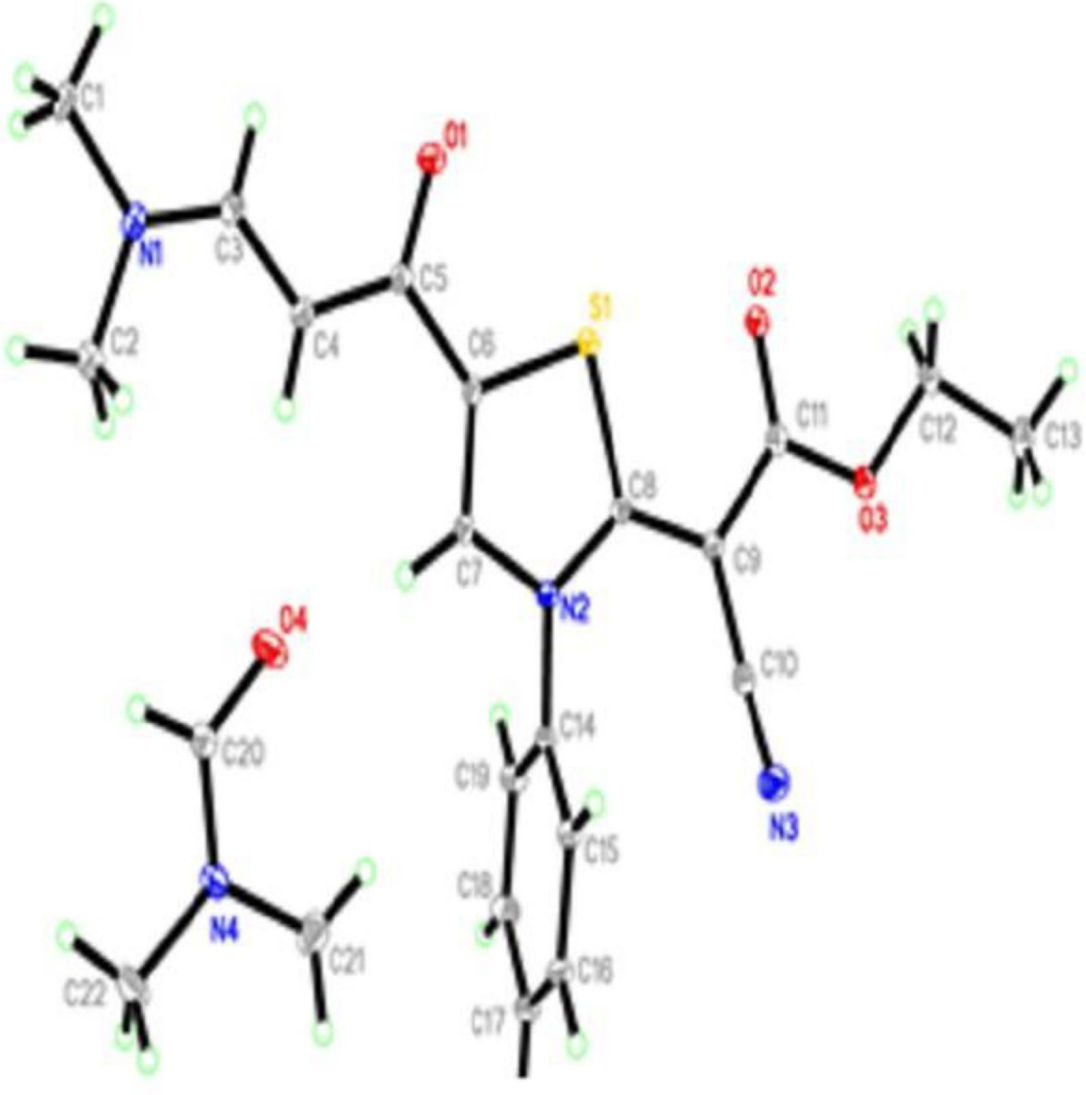
ORTEP diagram of thiazole **11**. Displacement ellipsoids are plotted at the 40% probability level for non-H atoms

**Fig. 4:**
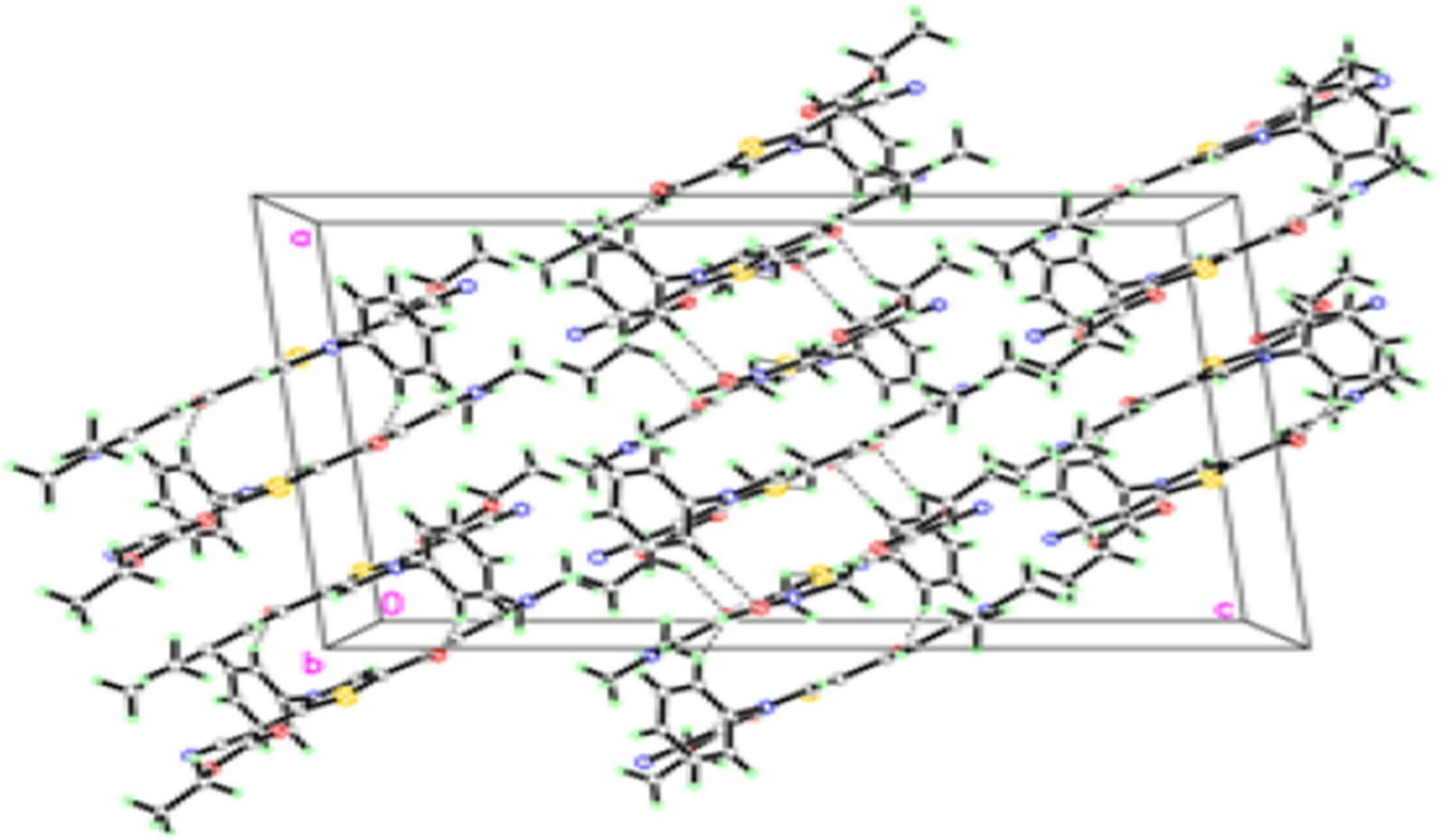
Molecular packing of thiazole **11** viewed hydrogen bonds which are drawn as dashed lines

Next, fusion of thiazole **6** with DMF-DMA in presence of DMF afforded the unexpected thiazole derivative **11** (Scheme 2). The structure of the isolated product was elucidated based on its elemental and spectral analysis (IR, NMR, MS and X-ray) (see "Experimental section") (Figs. 3, 4).

In many reports dimethylformamide were used as a formylating agent for indole [25], thiophene [26], and substituted benzene [27]. Based on these information, we suggested that the reaction was started via formylation of thiazole derivative **6** by DMF to afford the formyl derivative **7**, which involved a reversible opening of the thiazole ring to give intermediate **8**. The subsequent cyclization of **8** afforded **9**, which underwent dehydration to give the methyl ketone **10**. Reaction of intermediate **10** with dimethylformamide-dimethylacetal (DMF-DMA) afforded the unexpected thiazole derivative **11** (Scheme 2).

For more details see (Additional file 1: Tables S1–S6) (these files are available in the ESI section).

### Geometry optimization

**Fig. 5:**
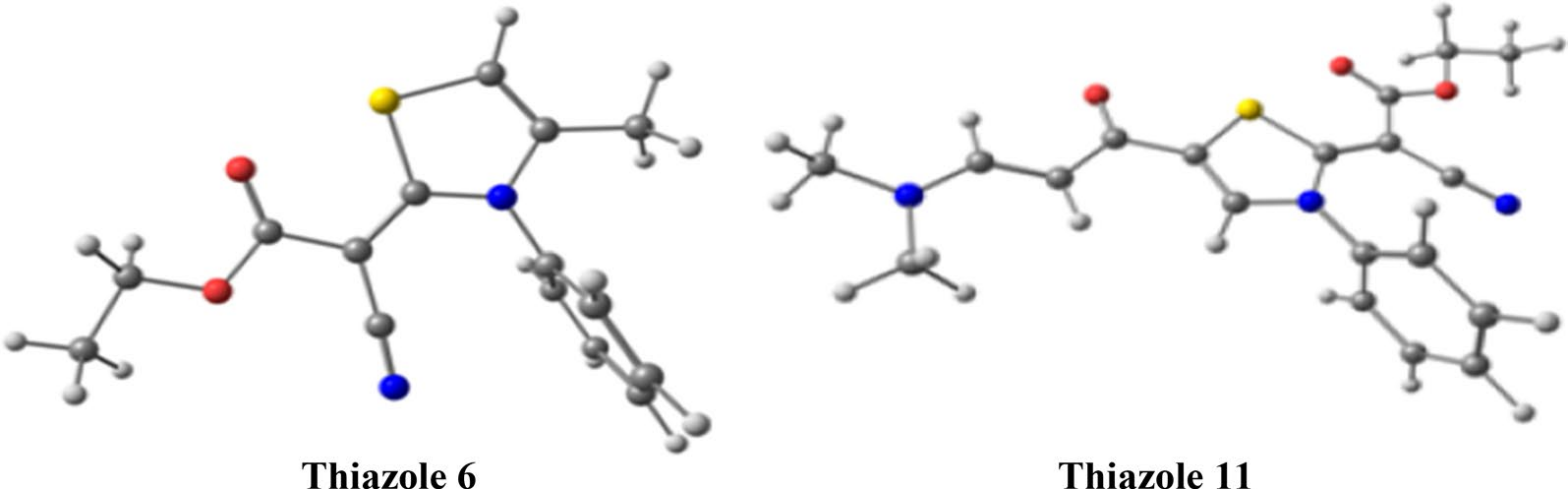
The optimized structure of the thiazoles **6** and **11**

**Fig. 6:**
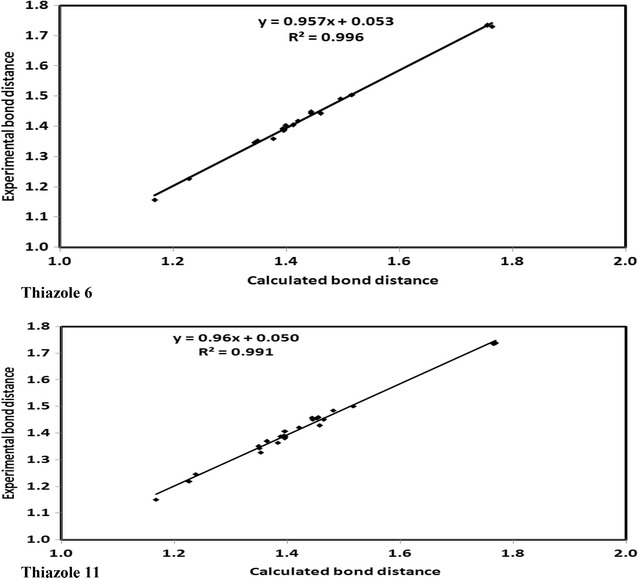
The correlations between the calculated and experimental bond distances of the thiazoles **6** and **11**

The optimized molecular geometries of the thiazole derivatives **6** and **11** are shown in Fig. 5 and the results of the calculated bond distances and angles are given in Additional file 1: Table S7. Good correlations were obtained between the calculated and experimental bond distances with correlation coefficients ranging from 0.991 to 0.996 (Fig. 6). The maximum differences between the calculations and experiments not exceed 0.03 Å for both compounds indicating the well prediction of the molecular geometries.

### Charge population analysis

**Fig. 7:**
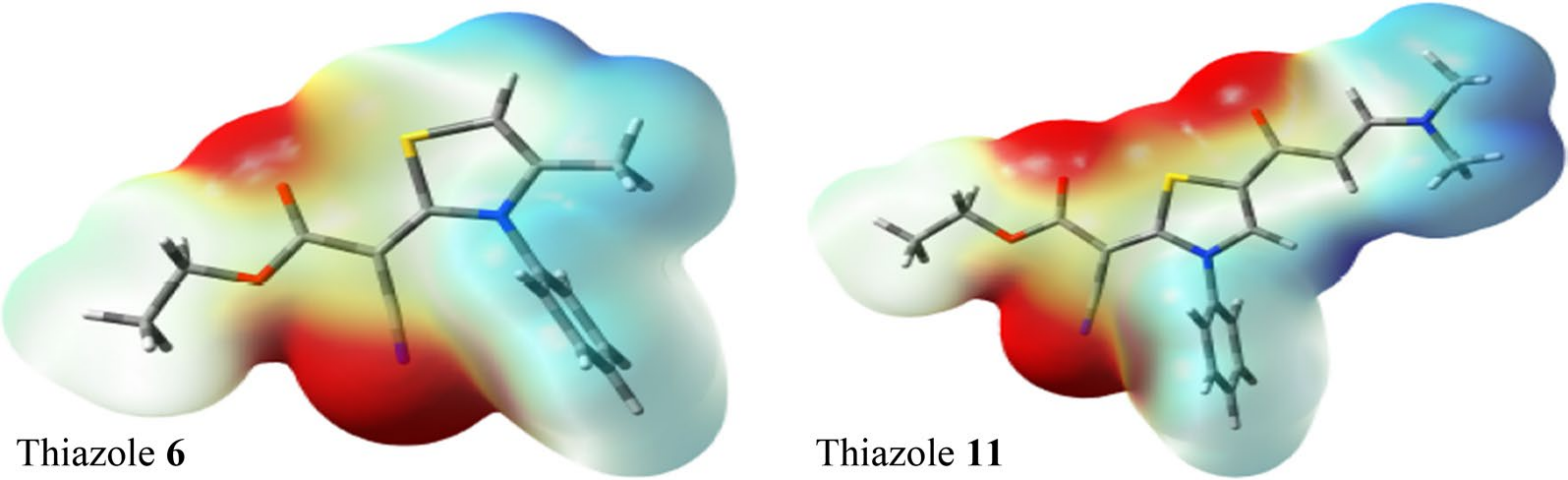
The MEP figure of the thiazoles **6** and **11**

The natural population analysis is performed to predict the natural charges (NC) at the different atomic sites (Additional file 1: Table S8). The ring sulphur atom has natural charge of 0.5079 and 0.5499e for thiazole **6** and thiazole **11**, respectively. In both cases, the S-atoms have electropositive nature where higher positive charge is found in thiazole **11** probably due to the presence of carbonyl group as electron withdrawing group directly attached to the ring while in thiazole **6**, there is one methyl as electron releasing group via inductive effect attached to the ring. The negative sites are related to the nitrogen and oxygen sites as also further confirmed from the molecular electrostatic potential (MEP) maps shown in Fig. 7.

### Frontier molecular orbitals

**Fig. 8:**
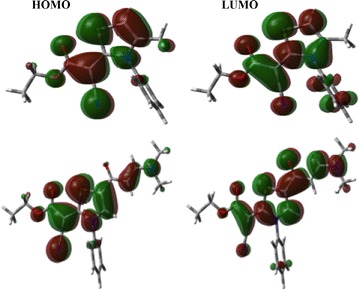
The frontier molecular orbitals of the synthesized compounds **6** and **11** calculated at the B3LYP/6-31G(d,p) level

The HOMO and LUMO levels of the thiazole derivatives **6** and **11** are shown in Fig. 8. The HOMO and LUMO energies of thiazole **6** are − 5.3582 and − 0.8765 eV, respectively while for thiazole **11** are − 5.3210 and − 1.5715 eV, respectively. As a result, the HOMO–LUMO energy gap is calculated to be 4.4818 and 3.7495 eV for compounds **6** and **11**, respectively. The HOMO and LUMO are mainly localized over the thiophene ring, C≡N and C=O groups for both compounds. Since the HOMO and LUMO levels are mainly located over the π-system of the studied compound so the HOMO–LUMO intramolecular charge transfer is mainly a π–π* transition.

### Cytotoxic activity

The anti-cancer activity of the thiazole derivatives **6** and **11** was determined against the Human Colon Carcinoma (HCT-116) cell line in comparison with the anticancer drug vinblastine, using MTT assay [28, 29]. The cytotoxic activity was expressed as the mean IC_50_ (the concentration of the test compounds required to kill half of the cell population) of three independent experiments (Table 1). The results revealed that thiazole **11** has moderate anticancer activity against colon carcinoma (HCT-116), while thiazole **6** has less activity.

**Table 1:** Viability values and IC_50_ of thiophenes **6** and **11** against HCT-116 Cell Line

S. no	Sample concentration (μg/mL) viability %							
	50	25	12.5	6.25	3.125	1.56	0	IC_50_ (μg)
Ref. D.	23.08	27.35	43.59	53.85	69.23	82.54	100	5.38
6	39.43	58.15	79.51	86.42	92.63	96.47	100	35.9
11	23.81	42.96	60.34	74.89	86.93	94.57	100	19.9

*Ref. D*. reference drug (Vinblastine), *S. No* sample number

## Experimental section

### Chemistry

#### General

All the melting points were measured on a Gallen Kamp apparatus in open glass capillaries and are uncorrected. The IR Spectra were recorded using Nicolet 6700 FT-IR spectrophotometer. ^1^H- and ^13^C-NMR spectra were recorded on a JEOL ECP 400 NMR spectrometer operating at 400 MHz in deuterated chloroform (CDCl_3_) as solvent and TMS as an internal standard; chemical shifts δ are expressed in ppm units. Mass spectra were recorded on a Shimadzu GCMS-QP 1000 EX mass spectrometer (Tokyo, Japan) at 70 eV. Elemental analysis was carried out on a 2400 CHN Elemental Analyzer. The single-crystal X-ray diffraction measurements were accomplished on a Bruker SMART APEX II CCD diffractometer. The biological evaluations of the products were carried out in the Medical Mycology Laboratory of the Regional Center for Mycology and Biotechnology of Al-Azhar University, Cairo, Egypt.

#### Synthesis of (Z)-ethyl 2-cyano-2-(4-methyl-3-phenylthiazol-2(3H)-ylidene)acetate (6)

### Method A

To a stirred solution of ethyl cyanoacetate (1.13 g, 1.07 mL, 10 mmol), in dimethylformamide (10 mL) was added potassium carbonate (1.38 g, 10 mmol). Stirring was continued at room temperature for 30 min, then phenylisothiocyanate (1.35 g, 1.2 mL, 10 mmol) was added dropwise to this mixture and stirring was continued for another 1 h. To this reaction mixture, chloroacetone (0.92 g, 0.8 mL, 10 mmol) was added and the mixture was stirred for additional 3 h at room temperature. Finally, the content was poured on cold water (50 mL). The crude solid product was filtered off and recrystallized from DMF, yield 85%, mp. 215 °C [lit mp [23]. 190 °C]; IR (KBr)v_max_1680 (CO), 2214 (C≡N), 2988 (aliphatic, CH), 3281(aromatic, CH) cm^−1^; ^1^H NMR (400 MHz, CDCl_3_): δ 1.19 (t, 3H. CH_3_, *J *= 7.2 Hz), 1.84 (s, 3H, CH_3_), 4.15 (q, 2H, CH_2_, *J *= 7.2 Hz), 6.39 (s, 1H. 5-H), 7.20–7.55 (m, 5H, Ar–H); ^13^C NMR (100 MHz, CDCl_3_): δ 14.46, 29.59, 60.48, 66.36, 105.62, 115.22, 128.72, 129.88, 131.07, 136.26, 138.45, 167.94, 168.05. Anal. calcd. for C_15_H_14_N_2_O_2_S: C, 62.92; H, 4.93; N, 9.78 Found: C, 62.89; H, 4.88; N, 9.79.

### Method B

A mixture of ethyl cyanoacetate (1.13 g, 1.07 mL, 10 mmol) in sodium ethoxide (0.23 g Sodium in 10 ml of absolute ethanol) was stirred for 10 min. To this mixture, phenyl isothiocyanate (1.35 g, 10 mmol) was added dropwise and the mixture was stirred for another 1 h. Chloroacetone (0.92 g, 0.8 mL, 10 mmol) was added to the reaction mixture and stirring was continued for 3 h. Finally, it was poured on cold water and the solid precipitate that formed was filtered and recrystallized from DMF to afford the same product which obtained from method A, yield 65%.

#### Synthesis of (Z)-ethyl 2-cyano-2-(5-((E)-3-(dimethylamino)acryloyl)-3-phenyl thiazol-2(3H)-ylidene)acetate (11)

A mixture of thiazole **6** (2.86 g, 10 mmol) and DMF-DMA (1.19 g, 1.33 mL, 10 mmol) in DMF (3 mL) was heated on a water bath for 1 h, then left to cool to room temperature. The precipitated solid filtered off, washed with EtOH and recrystallized from DMF to afford the thiazole derivative **11** in 82% yield, m.p. 260 °C; IR (KBr) v_max_ 1669 (C=O), 2189 (C≡N), 2928 (aliphatic, CH), 3056 (aromatic, CH) cm^−1^; ^1^H NMR (400 MHz, CDCl_3_): δ 1.26 (t, 3H. CH_3_, *J *= 7.3 Hz), 2.88 (s, 3H, CH_3_), 3.16 (s, 3H, CH_3_), 4.21 (q, 2H, CH_2_, *J *= 7.3 Hz), 5.28 (d, 1H, CH, *J* = 12.5 Hz), 7.43–7.56 (m, 7H, Ar–H); MS *m/z* (%) 369 (M^+^, 23.78), 299 (0.98), 271(1.36), 98 (100), 77 (10.05), 70 (7.8). calcd. for C_19_H_19_N_3_O_3_S: C, 61.77; H, 5.18; N, 11.37. Found: 61.82; H, 5.21; N, 11.28.

### X-Ray analysis

The thiazoles of **6** and **11** were obtained as single crystals by slow evaporation from DMF solution of the pure compound at room temperature. Data were collected on a BrukerAPEX-II D8 Venture area diffractometer, equipped with graphite monochromatic Mo *K*α radiation, λ = 0.71073 Å at 100 (2) K. Cell refinement and data reduction were carried out by Bruker SAINT. SHELXT [30, 31] was used to solve structure. The final refinement was carried out by full-matrix least-squares techniques with anisotropic thermal data for nonhydrogen atoms on* F*. CCDC 1504892 and 1505279 contain the supplementary crystallographic data for this compound can be obtained free of charge from the Cambridge Crystallographic Data Centre via www.ccdc.cam.ac.uk/data_request/cif.

### Computational details

The X-ray structure coordinates of the studied thiazoles were used for geometry optimization followed by frequency calculations. For this task, we used Gaussian 03 software [32] and B3LYP/6‒31G(d,p) method. All obtained frequencies are positive, and no imaginary modes were detected. GaussView4.1 [33] and Chemcraft [34] programs have been used to extract the calculation results and to visualize the optimized structures.

### Cytotoxic activity

The cytotoxic activity of the synthesized compounds was determined against Human Colon Carcinoma (HCT-116) by the standard MTT assay [28, 29].

## Conclusions

Stereoselective synthesis of (Z)-ethyl 2-cyano-2-(4-methyl-3-phenylthiazol-2(3H)-ylidene) acetate (**6**) and its unexpected reaction with DMF-DMA gave (Z)-ethyl 2-cyano-2-(5-((E)-3-(dimethylamino)acryloyl)-3-phenylthiazol-2(3H)-ylidene)acetate (**11**). Optimized molecular structures at the B3LYP/6-31G(d,p) level are presented. Thiazole **11** has more electropositive S-atom than Thiazole **6**. The HOMO–LUMO energy gap is lower in the former compared to the latter. The cytotoxic activity of the synthesized thiazoles was evaluated and the results revealed that thiazole derivative **11** had more activity than thiazole derivative **6**.

## Additional file

**Additional file 1: Table S1.** The crystal and experimental data of thiazole **6. Table S2.** Selected geometric parameters (Å, °) of thiazole **6. Table S3.** Hydrogen-bond geometry (Å, °) of thiazole **6. Table S4.** The crystal and experimental data of thiazole **11**. **Table S5.** Selected geometric parameters (Å, °) thiazole **11**. **Table S6.** Hydrogen-bond geometry (Å, °) thiazole **11**. **Figure S1.** The atom numbering scheme of the optimized molecular structures of the studied molecules. **Table S7.** The experimental and calculated geometric parameters of the studied molecules. **Table S8.** The natural atomic charges of the studied systems using B3LYP method.
