# Supplementary material for: Stereoselective synthesis, X-ray analysis, computational studies and biological evaluation of new thiazole derivatives as potential anticancer agents
Source: Chem Cent J. 2018 May 11;12:56. doi: 10.1186/s13065-018-0420-7 (PMC5945573; doi:10.1186/s13065-018-0420-7)
Supplement: Supplementary file 1 — Additional file 1: Table S1. The crystal and experimental data of thiazole 6. Table S2. Selected geometric parameters (Å, °) of thiazole 6. Table S3. Hydrogen-bond geometry (Å, °) of thiazole 6. Table S4. The crystal and experimental data of thiazole 11. Table S5. Selected geometric parameters (Å, °) thiazole 11. Table S6. Hydrogen-bond geometry (Å, °) thiazole 11. Figure S1. The atom numbering scheme of the optimized molecular structures of the studied molecules. Table S7. The experimental and calculated geometric parameters of the studied molecules. Table S8. The natural atomic charges of the studied systems using B3LYP method. [file 13065_2018_420_MOESM1_ESM.docx]

Stereoselective synthesis, X-ray analysis, computational studies and biological evaluation of new thiazole derivatives as potential anticancer agents

Yahia N. Mabkhot^1,*^, Mohammed M. Alharbi^1^, Salim. S. Al-Showiman^1^, Hazem A. Ghabbour^2,3^, Nabila A. Kheder^4,5^, Saied M. Soliman^6,7^ and Wolfgang Frey^8^

**Table S1**. The crystal and experimental data of thiazole **6**

| **Crystal data** | |
| --- | --- |
| Chemical formula | [C_15_H_14_N_2_O_2_S](file:///C:\Users\HP\Downloads\a%20_chemical_formula_sum) |
| *M*r | [286.34](file:///C:\Users\HP\Downloads\a%20_chemical_formula_weight) |
| Crystal system, space group | [Triclinic](file:///C:\Users\HP\Downloads\a%20_symmetry_cell_setting), [*P*](file:///C:\Users\HP\Downloads\a%20_symmetry_space_group_name_H-M)-1 |
| Temperature (K) | [100](file:///C:\Users\HP\Downloads\a%20_cell_measurement_temperature) |
| a, b, c (Å) | [9.0547 (5)](file:///C:\Users\HP\Downloads\a%20_cell_length_a), [9.3132 (6)](file:///C:\Users\HP\Downloads\a%20_cell_length_b), [9.6414 (6)](file:///C:\Users\HP\Downloads\a%20_cell_length_c) |
| α, β, γ (°) | [87.707 (2)](file:///C:\Users\HP\Downloads\a%20_cell_angle_alpha), [62.521 (2)](file:///C:\Users\HP\Downloads\a%20_cell_angle_beta), [75.065 (2)](file:///C:\Users\HP\Downloads\a%20_cell_angle_gamma) |
| V (Å^3^) | [693.99 (7)](file:///C:\Users\HP\Downloads\a%20_cell_volume) |
| Z | [2](file:///C:\Users\HP\Downloads\a%20_cell_formula_units_Z) |
| Radiation type | [Mo K*α*](file:///C:\Users\HP\Downloads\a%20_diffrn_radiation_type) |
| µ (mm^−1^) | [0.24](file:///C:\Users\HP\Downloads\a%20_exptl_absorpt_coefficient_mu) |
| Crystal size (mm) | [0.78](file:///C:\Users\HP\Downloads\a%20_exptl_crystal_size_max) × [0.69](file:///C:\Users\HP\Downloads\a%20_exptl_crystal_size_mid) × [0.56](file:///C:\Users\HP\Downloads\a%20_exptl_crystal_size_min) |
| **Data collection** | |
| Diffractometer | [Bruker APEX-II CCD  diffractometer](file:///C:\Users\HP\Downloads\a%20_diffrn_measurement_device_type) |
| Absorption correction | [Multi-scan](file:///C:\Users\HP\Downloads\a%20_exptl_absorpt_correction_type) [SADABS Bruker 2014](file:///C:\Users\HP\Downloads\a%20_exptl_absorpt_process_details) |
| T_min_, T_max_ | [0.838](file:///C:\Users\HP\Downloads\a%20_exptl_absorpt_correction_T_min), [0.880](file:///C:\Users\HP\Downloads\a%20_exptl_absorpt_correction_T_max) |
| No. of measured, independent and observed [[I> 2σ(I)](file:///C:\Users\HP\Downloads\a%20_reflns_threshold_expression)] reflections | [33062](file:///C:\Users\HP\Downloads\a%20_diffrn_reflns_number), [5294](file:///C:\Users\HP\Downloads\a%20_reflns_number_total), [4698](file:///C:\Users\HP\Downloads\a%20_reflns_number_gt) |
| R_int_ | [0.053](file:///C:\Users\HP\Downloads\a%20_diffrn_reflns_av_R_equivalents) |
| **Refinement** | |
| R[*F*^2^> 2σ(*F*^2^)], wR(*F*^2^), S | [0.036](file:///C:\Users\HP\Downloads\a%20_refine_ls_R_factor_gt), [0.104](file:///C:\Users\HP\Downloads\a%20_refine_ls_wR_factor_ref), [1.09](file:///C:\Users\HP\Downloads\a%20_refine_ls_goodness_of_fit_ref) |
| No. of reflections | [5294](file:///C:\Users\HP\Downloads\a%20_refine_ls_number_reflns) |
| No. of parameters | [183](file:///C:\Users\HP\Downloads\a%20_refine_ls_number_parameters) |
| No. of restraints | [0](file:///C:\Users\HP\Downloads\a%20_refine_ls_number_restraints) |
| H-atom treatment | [H atoms treated by a mixture of independent and constrained refinement](file:///C:\Users\HP\Downloads\a%20_refine_ls_hydrogen_treatment) |
| Δρ_max_, Δρ_min_ (e Å^−3^) | [0.55](file:///C:\Users\HP\Downloads\a%20_refine_diff_density_max), [−0.55](file:///C:\Users\HP\Downloads\a%20_refine_diff_density_min) |

[**Table S2**.Selected geometric parameters (Å, °)](file:///M:\cmos\yahia\y63\a%20_geom_bond_distance) of thiazole **6**

| S1—C8 | 1.7353 (11) | N1—C6 | 1.4439 (12) |
| --- | --- | --- | --- |
| S1—C9 | 1.7307 (10) | N1—C7 | 1.4052 (15) |
| O1—C11 | 1.2275 (13) | N1—C9 | 1.3588 (12) |
| O2—C11 | 1.3531 (14) | N2—C14 | 1.1570 (17) |
| O2—C12 | 1.4483 (13) |  |  |
| C8—S1—C9 | 91.48 (5) | S1—C8—C7 | 111.96 (9) |
| C11—O2—C12 | 115.45 (8) | S1—C9—N1 | 109.61 (8) |
| C6—N1—C7 | 120.64 (8) | S1—C9—C10 | 123.56 (6) |
| C6—N1—C9 | 123.37 (9) | N1—C9—C10 | 126.83 (9) |
| C7—N1—C9 | 115.18 (8) | O1—C11—O2 | 122.44 (9) |
| N1—C6—C1 | 119.85 (8) | O1—C11—C10 | 124.66 (10) |
| N1—C6—C5 | 117.99 (8) | O2—C11—C10 | 112.90 (9) |
| N1—C7—C8 | 111.76 (9) | O2—C12—C13 | 107.33 (9) |
| N1—C7—C15 | 119.84 (9) | N2—C14—C10 | 175.54 (11) |

[**Table S3**.Hydrogen-bond geometry (Å, °)](file:///M:\cmos\yahia\y63\a%20_geom_hbond_atom_site_label_D)of thiazole **6**

| D—H···A | D—H | H···A | D···A | D—H···A |
| --- | --- | --- | --- | --- |
| C4—H4A···O1i | 0.9500 | 2.5300 | 3.3371(13) | 142.00 |
| C5—H5A···O1ii | 0.9500 | 2.5900 | 3.4387(13) | 148.00 |
| C15—H15A···N2iii | 0.9800 | 2.5200 | 3.4438(18) | 157.00 |
| Symmetry codes: (i) x, y+1, z; (ii) −x, −y+1, −z+2; (iii) x−1, y, z. | | | | |

**Table S4**. The crystal and experimental data of thiazole **11**.

| **Crystal data** | |
| --- | --- |
| Chemical formula | [C_22_H_26_N_4_O_4_S](file:///C:\Users\HP\Downloads\a_a%20_chemical_formula_sum) |
| Mr | [442.53](file:///C:\Users\HP\Downloads\a_a%20_chemical_formula_weight) |
| Crystal system, space group | [Monoclinic](file:///C:\Users\HP\Downloads\a_a%20_symmetry_cell_setting), [C2/c](file:///C:\Users\HP\Downloads\a_a%20_symmetry_space_group_name_H-M) |
| Temperature (K) | [100](file:///C:\Users\HP\Downloads\a_a%20_cell_measurement_temperature) |
| a, b, c (Å) | [14.963 (4)](file:///C:\Users\HP\Downloads\a_a%20_cell_length_a), [11.979 (3)](file:///C:\Users\HP\Downloads\a_a%20_cell_length_b), [24.902 (8)](file:///C:\Users\HP\Downloads\a_a%20_cell_length_c) |
| β (°) | [97.012 (11)](file:///C:\Users\HP\Downloads\a_a%20_cell_angle_beta) |
| V (Å^3^) | [4430 (2)](file:///C:\Users\HP\Downloads\a_a%20_cell_volume) |
| Z | [8](file:///C:\Users\HP\Downloads\a_a%20_cell_formula_units_Z) |
| Radiation type | [Mo Kα](file:///C:\Users\HP\Downloads\a_a%20_diffrn_radiation_type) |
| µ (mm−1) | [0.18](file:///C:\Users\HP\Downloads\a_a%20_exptl_absorpt_coefficient_mu) |
| Crystal size (mm) | [0.64](file:///C:\Users\HP\Downloads\a_a%20_exptl_crystal_size_max) × [0.41](file:///C:\Users\HP\Downloads\a_a%20_exptl_crystal_size_mid) × [0.35](file:///C:\Users\HP\Downloads\a_a%20_exptl_crystal_size_min) |
| **Data collection** | |
| Diffractometer | [Bruker APEX-II CCD  diffractometer](file:///C:\Users\HP\Downloads\a_a%20_diffrn_measurement_device_type) |
| Absorption correction | [Multi-scan](file:///C:\Users\HP\Downloads\a_a%20_exptl_absorpt_correction_type) [SADABS Bruker 2014](file:///C:\Users\HP\Downloads\a_a%20_exptl_absorpt_process_details) |
| T_min_, T_max_ | [0.776](file:///C:\Users\HP\Downloads\a_a%20_exptl_absorpt_correction_T_min), [0.814](file:///C:\Users\HP\Downloads\a_a%20_exptl_absorpt_correction_T_max) |
| No. of measured, independent and observed [[I> 2σ(I)](file:///C:\Users\HP\Downloads\a_a%20_reflns_threshold_expression)] reflections | [34998](file:///C:\Users\HP\Downloads\a_a%20_diffrn_reflns_number), [4756](file:///C:\Users\HP\Downloads\a_a%20_reflns_number_total), [4277](file:///C:\Users\HP\Downloads\a_a%20_reflns_number_gt) |
| R_int_ | [0.043](file:///C:\Users\HP\Downloads\a_a%20_diffrn_reflns_av_R_equivalents) |
| Refinement | |
| R[*F*^2^> 2σ(*F*^2^)], wR(*F*^2^), S | [0.035](file:///C:\Users\HP\Downloads\a_a%20_refine_ls_R_factor_gt), [0.099](file:///C:\Users\HP\Downloads\a_a%20_refine_ls_wR_factor_ref), [1.00](file:///C:\Users\HP\Downloads\a_a%20_refine_ls_goodness_of_fit_ref) |
| No. of reflections | [4756](file:///C:\Users\HP\Downloads\a_a%20_refine_ls_number_reflns) |
| No. of parameters | [286](file:///C:\Users\HP\Downloads\a_a%20_refine_ls_number_parameters) |
| No. of restraints | [0](file:///C:\Users\HP\Downloads\a_a%20_refine_ls_number_restraints) |
| H-atom treatment | [H atoms treated by a mixture of independent and constrained refinement](file:///C:\Users\HP\Downloads\a_a%20_refine_ls_hydrogen_treatment) |
| Δρ_max_, Δρ_min_ (e Å^−3^) | [0.64](file:///C:\Users\HP\Downloads\a_a%20_refine_diff_density_max), [−0.41](file:///C:\Users\HP\Downloads\a_a%20_refine_diff_density_min) |

[**Table S5**.Selected geometric parameters (Å, °)](file:///M:\cmos\yahia\y63\a_a%20_geom_bond_distance) thiazole **11**.

| S1—C6 | 1.7381 (14) | N1—C3 | 1.3283 (17) |
| --- | --- | --- | --- |
| S1—C8 | 1.7351 (14) | N2—C8 | 1.3637 (18) |
| O1—C5 | 1.2458 (17) | N2—C14 | 1.4511 (17) |
| O2—C11 | 1.2192 (17) | N2—C7 | 1.3883 (17) |
| O3—C11 | 1.3512 (16) | N3—C10 | 1.1511 (19) |
| O3—C12 | 1.4577 (18) | N4—C20 | 1.3261 (19) |
| O4—C20 | 1.2288 (18) | N4—C21 | 1.451 (2) |
| N1—C1 | 1.4557 (19) | N4—C22 | 1.456 (2) |
| N1—C2 | 1.4593 (17) |  |  |
| C6—S1—C8 | 91.36 (6) | S1—C6—C7 | 111.12 (10) |
| C11—O3—C12 | 113.92 (11) | S1—C6—C5 | 116.72 (10) |
| C1—N1—C2 | 117.64 (11) | N2—C7—C6 | 113.32 (12) |
| C1—N1—C3 | 120.59 (11) | S1—C8—C9 | 122.57 (10) |
| C2—N1—C3 | 121.76 (12) | S1—C8—N2 | 109.74 (9) |
| C7—N2—C8 | 114.46 (10) | N2—C8—C9 | 127.69 (12) |
| C7—N2—C14 | 119.02 (11) | N3—C10—C9 | 175.47 (15) |
| C8—N2—C14 | 126.23 (10) | O2—C11—C9 | 123.90 (12) |
| C21—N4—C22 | 117.52 (13) | O2—C11—O3 | 122.65 (13) |
| C20—N4—C21 | 122.21 (13) | O3—C11—C9 | 113.45 (11) |
| C20—N4—C22 | 120.27 (13) | O3—C12—C13 | 107.50 (12) |
| N1—C3—C4 | 126.87 (12) | N2—C14—C15 | 118.06 (11) |
| O1—C5—C4 | 125.19 (12) | N2—C14—C19 | 119.69 (11) |
| O1—C5—C6 | 116.36 (11) | O4—C20—N4 | 125.40 (14) |

[**Table S6**.](file:///M:\cmos\yahia\y63\a_a%20_geom_hbond_atom_site_label_D) [Hydrogen-bond geometry (Å, °)](file:///M:\cmos\yahia\y63\a_a%20_geom_hbond_atom_site_label_D) thiazole **11**.

| D—H···A | D—H | H···A | D···A | D—H···A |
| --- | --- | --- | --- | --- |
| C4—H4A···O4i | 0.9300 | 2.3900 | 3.301(2) | 167.00 |
| C7—H7A···O4i | 0.9300 | 2.2700 | 3.154(2) | 158.00 |
| C12—H12B···O1ii | 0.9700 | 2.4800 | 3.318(2) | 144.00 |
| C15—H15A···O1iii | 0.9300 | 2.3700 | 3.240(2) | 155.00 |
| C19—H19A···O4iv | 0.9300 | 2.5400 | 3.397(2) | 154.00 |
| Symmetry codes: (i) x−1/2, −y−1/2, z−1/2; (ii) −x−3/2, −y+1/2, −z; (iii) −x−1, −y, −z; (iv) −x−1, y, −z+1/2. | | | | |
|  | | | | |
|  | | | | |
|  | | | | |

| 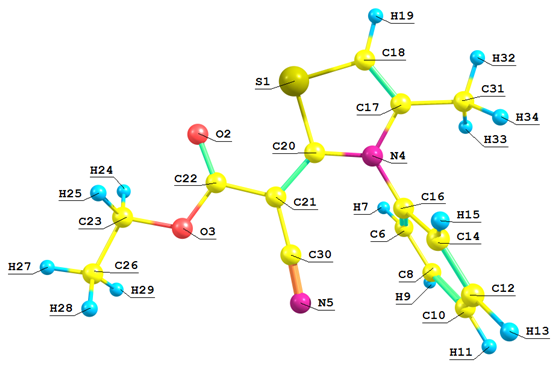**Thiazole 6** |
| --- |
| 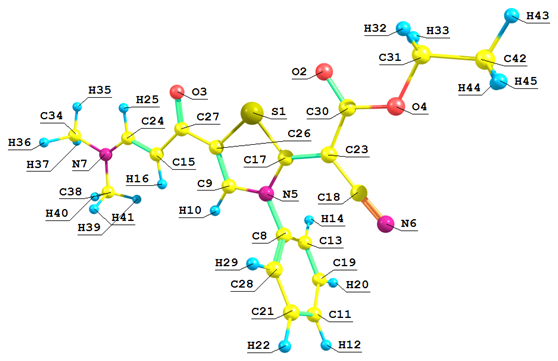**Thiazole 11**. |
| **Fig. S1** The atom numbering scheme of the optimized molecular structures of the studied molecules. |

**Table S7**. The experimental and calculated geometric parameters of the studied molecules.

|  | Calc. | X-ray |  | Calc. | X-ray |
| --- | --- | --- | --- | --- | --- |
|  | Thiazole **6** | |  | Thiazole **11** | |
| R(1-18) | 1.755 | 1.735 | R(1-17) | 1.765 | 1.735 |
| R(1-20) | 1.764 | 1.731 | R(1-26) | 1.769 | 1.738 |
| R(2-22) | 1.228 | 1.227 | R(2-30) | 1.226 | 1.219 |
| R(3-22) | 1.349 | 1.353 | R(3-27) | 1.238 | 1.246 |
| R(3-23) | 1.444 | 1.448 | R(4-30) | 1.349 | 1.351 |
| R(4-16) | 1.444 | 1.444 | R(4-31) | 1.444 | 1.458 |
| R(4-17) | 1.412 | 1.405 | R(5-8) | 1.445 | 1.451 |
| R(4-20) | 1.377 | 1.359 | R(5-9) | 1.388 | 1.388 |
| R(5-30) | 1.167 | 1.157 | R(5-17) | 1.383 | 1.364 |
| R(6-8) | 1.394 | 1.393 | R(6-18) | 1.167 | 1.151 |
| R(6-16) | 1.395 | 1.388 | R(7-24) | 1.353 | 1.328 |
| R(8-10) | 1.396 | 1.39 | R(7-34) | 1.455 | 1.459 |
| R(10-12) | 1.396 | 1.391 | R(7-38) | 1.453 | 1.456 |
| R(12-14) | 1.394 | 1.392 | R(8-13) | 1.395 | 1.381 |
| R(14-16) | 1.395 | 1.387 | R(8-28) | 1.395 | 1.388 |
| R(17-18) | 1.345 | 1.346 | R(9-26) | 1.35 | 1.344 |
| R(17-31) | 1.496 | 1.492 | R(11-19) | 1.396 | 1.388 |
| R(20-21) | 1.399 | 1.404 | R(11-21) | 1.396 | 1.386 |
| R(21-22) | 1.461 | 1.443 | R(13-19) | 1.394 | 1.39 |
| R(21-30) | 1.421 | 1.418 | R(15-24) | 1.364 | 1.37 |
| R(23-26) | 1.516 | 1.505 | R(15-27) | 1.457 | 1.429 |
|  |  |  | R(17-23) | 1.396 | 1.407 |
|  |  |  | R(18-23) | 1.421 | 1.422 |
|  |  |  | R(21-28) | 1.394 | 1.391 |
|  |  |  | R(23-30) | 1.464 | 1.451 |
|  |  |  | R(26-27) | 1.481 | 1.485 |
|  |  |  | R(31-42) | 1.517 | 1.501 |

Full details regarding geometric parameters could be obtained with the aid of Cartesian coordinates given below. Atom numbering refers to the structures above.

Cartesian coordinated of the optimized geometry of thiazole **6**

S -0.525664000 2.660614000 -0.000202000

O -2.844255000 1.463664000 0.000576000

O -3.272925000 -0.759722000 -0.000467000

N 1.290155000 0.835313000 -0.000318000

N -0.513545000 -2.608830000 -0.004252000

C 2.349943000 -1.014090000 -1.213869000

H 2.059409000 -0.540248000 -2.145942000

C 3.050736000 -2.218927000 -1.208748000

H 3.316981000 -2.690355000 -2.149384000

C 3.397670000 -2.821646000 0.001302000

H 3.936307000 -3.764132000 0.001737000

C 3.049032000 -2.218810000 1.210806000

H 3.313978000 -2.690141000 2.151857000

C 2.348254000 -1.013961000 1.214835000

H 2.056470000 -0.539986000 2.146456000

C 1.995857000 -0.424519000 0.000203000

C 1.996352000 2.058055000 0.000075000

C 1.168652000 3.117848000 0.000104000

H 1.455415000 4.158974000 0.000281000

C -0.081359000 0.953394000 -0.000527000

C -1.030945000 -0.074373000 -0.000858000

C -2.442438000 0.303152000 -0.000162000

C -4.684622000 -0.458033000 0.000244000

H -4.920300000 0.145125000 -0.882481000

H -4.919035000 0.146643000 0.882246000

C -5.427283000 -1.780138000 0.001787000

H -6.506770000 -1.599413000 -0.000524000

H -5.176206000 -2.367498000 0.889237000

H -5.173009000 -2.370929000 -0.882477000

C -0.712476000 -1.459364000 -0.001671000

C 3.492665000 2.068051000 0.000238000

H 3.846121000 3.100687000 0.000521000

H 3.901161000 1.562568000 -0.880785000

H 3.901050000 1.562131000 0.881051000

Cartesian coordinated of the optimized geometry of thiazole **11**.

S 0.490812000 1.033129000 -0.078127000

O 2.203395000 3.051435000 -0.077221000

O -2.375080000 0.578204000 -0.111510000

O 4.465697000 2.950888000 -0.050139000

N 1.891222000 -1.132622000 -0.000541000

N 5.622835000 -0.165942000 0.002992000

N -4.647062000 -2.937412000 -0.146134000

C 2.940598000 -2.123511000 0.069196000

C 0.563888000 -1.543213000 -0.024453000

H 0.373386000 -2.606553000 -0.002025000

C 4.892432000 -4.091104000 0.206793000

H 5.663489000 -4.853470000 0.260192000

C 3.423437000 -2.693302000 -1.108778000

H 3.042709000 -2.352625000 -2.066129000

C -2.497631000 -1.806096000 -0.106210000

H -1.912002000 -2.717584000 -0.102041000

C 2.064938000 0.237858000 -0.028924000

C 4.553147000 0.299450000 -0.008143000

C 4.406312000 -3.678906000 -1.034895000

H 4.796922000 -4.118322000 -1.947248000

C 4.392747000 -3.523470000 1.379910000

H 4.772360000 -3.842443000 2.345405000

C 3.279326000 0.929722000 -0.025467000

C -3.868693000 -1.837143000 -0.107958000

H -4.407677000 -0.893152000 -0.081839000

C -0.330637000 -0.533392000 -0.067530000

C -1.815294000 -0.530123000 -0.097635000

C 3.409062000 -2.538195000 1.315953000

H 3.015476000 -2.080138000 2.217484000

C 3.236691000 2.391393000 -0.053246000

C 4.501075000 4.393084000 -0.079685000

H 3.957358000 4.781177000 0.787738000

H 3.979974000 4.744324000 -0.976220000

C -6.096966000 -2.843833000 -0.010927000

H -6.398963000 -1.795917000 -0.017998000

H -6.431674000 -3.303395000 0.928623000

H -6.589349000 -3.366113000 -0.840039000

C -4.055819000 -4.264634000 -0.178788000

H -3.299321000 -4.326859000 -0.968329000

H -4.833507000 -5.001253000 -0.387896000

H -3.579122000 -4.522068000 0.776994000

C 5.959983000 4.807623000 -0.069712000

H 6.036255000 5.899323000 -0.092107000

H 6.485191000 4.405486000 -0.940415000

H 6.462336000 4.443404000 0.830661000

O -6.303198000 0.434585000 0.012699000

N -6.192458000 2.731016000 0.137519000

C -6.927005000 3.976185000 0.264358000

H -7.998311000 3.772393000 0.335909000

H -6.752860000 4.622268000 -0.604773000

H -6.616023000 4.522989000 1.162965000

C -6.831093000 1.537109000 0.118708000

H -7.928066000 1.649391000 0.211816000

C -4.740468000 2.816240000 0.026595000

H -4.311147000 1.818358000 -0.045335000

H -4.325331000 3.321571000 0.906141000

H -4.463548000 3.392517000 -0.863958000

**Table S8**. The natural atomic charges of the studied systems using B3LYP method.

|  | **Thiazole 6** |  | **Thiazole 11** |
| --- | --- | --- | --- |
| S1 | 0.5079 | S1 | 0.5499 |
| O2 | -0.6400 | O2 | -0.6310 |
| O3 | -0.5454 | O3 | -0.5991 |
| N4 | -0.4024 | O4 | -0.5464 |
| N5 | -0.3416 | N5 | -0.3951 |
| C6 | -0.2221 | N6 | -0.3395 |
| H7 | 0.2536 | N7 | -0.4116 |
| C8 | -0.2204 | C8 | 0.1172 |
| H9 | 0.2483 | C9 | -0.0004 |
| C10 | -0.2175 | H10 | 0.2539 |
| H11 | 0.2469 | C11 | -0.2187 |
| C12 | -0.2204 | H12 | 0.2466 |
| H13 | 0.2483 | C13 | -0.2200 |
| C14 | -0.2222 | H14 | 0.2544 |
| H15 | 0.2536 | C15 | -0.4566 |
| C16 | 0.1165 | H16 | 0.2221 |
| C17 | 0.1650 | C17 | 0.0951 |
| C18 | -0.4781 | C18 | 0.2662 |
| H19 | 0.2693 | C19 | -0.2213 |
| C20 | 0.1075 | H20 | 0.2481 |
| C21 | -0.3922 | C21 | -0.2216 |
| C22 | 0.8119 | H22 | 0.2480 |
| C23 | -0.1174 | C23 | -0.3854 |
| H24 | 0.2260 | C24 | 0.0824 |
| H25 | 0.2260 | H25 | 0.2509 |
| C26 | -0.7104 | C26 | -0.3238 |
| H27 | 0.2392 | C27 | 0.4865 |
| H28 | 0.2429 | C28 | -0.2205 |
| H29 | 0.2430 | H29 | 0.2541 |
| C30 | 0.2665 | C30 | 0.8122 |
| C31 | -0.7180 | C31 | -0.1177 |
| H32 | 0.2593 | H32 | 0.2267 |
| H33 | 0.2582 | H33 | 0.2267 |
| H34 | 0.2582 | C34 | -0.4716 |
|  |  | H35 | 0.2451 |
|  |  | H36 | 0.2263 |
|  |  | H37 | 0.2319 |
|  |  | C38 | -0.4870 |
|  |  | H39 | 0.2404 |
|  |  | H40 | 0.2396 |
|  |  | H41 | 0.2289 |
|  |  | C42 | -0.7104 |
|  |  | H43 | 0.2392 |
|  |  | H44 | 0.2426 |
|  |  | H45 | 0.2426 |
